# Local Welfare Systems and Health Inequalities: The Effects of Institutional Overlapping and Local Variations

**DOI:** 10.3390/ijerph192315447

**Published:** 2022-11-22

**Authors:** María Jesús Rodríguez-García, Clemente J. Navarro-Yáñez, Ángel R. Zapata-Moya

**Affiliations:** Center for Sociology and Urban Policies, Pablo de Olavide University, 41013 Sevilla, Spain

**Keywords:** local welfare system, health inequalities, urban policies, contextual analysis, Spain

## Abstract

A growing research agenda shows the importance of local welfare systems in understanding socio-spatial inequalities in health. Welfare services provided by local governments overlap with those provided by other levels of government. Thus, differences in the provision of welfare services between municipalities could explain differences in residents’ health, moderating the magnitude of health inequalities if local governments deploy actions capable of positively influencing the social determinants of health. This article attempts to analyse this idea in the Spanish case, exploring the influence of local policies according to the orientation of municipal spending on three indicators of the population’s health status: self-perceived health, healthy practices and activity limitations due to health problems. A multilevel cross-sectional study was designed using information from two waves of the 2006–2007 and 2011–2012 National Health Survey for the population aged 15 years and older (N = 31,378) residing in Spanish municipalities of 20,000 inhabitants or over (N = 373). The results show that the magnitude of inequalities in self-perceived health, in the adoption of healthy practices and in daily activity limitations by social class are smaller as municipalities” spending was oriented towards policy areas considered as redistributive. Therefore, the proposed institutional overlap thesis could help understand the role of subnational governments on the magnitude of health inequalities, as well as in comparative analysis between countries with institutional systems in which local governments have a greater or lesser capacity to provide welfare services.

## 1. Introduction

Even though seminal studies, such as those of Esping-Andersen or Ferrera [1,2,3], have already noted that research into welfare regimes should not be limited to national systems, analyses of welfare states and inequalities have focused mainly on national scales. Nevertheless, there is a growing research agenda on local welfare systems and their effects on socio-spatial inequalities. These works showed local variations in welfare service provision and their effects in some specific policy areas and inequality processes [4,5]. In addition to local socio-economic conditions, the ‘institutional imbrication’ of local governments in the multilevel governance system of each country set an opportunity structure for them to explain their actions; for instance, spending orientations (more or less redistributive spending) and alliances with other governmental and non-governmental actors (regional governments, other municipalities, civic associations, business, …) [6,7,8]. This institutional opportunity structure could explain differences in social inequalities among municipalities and contribute to explaining differences between countries [9,10,11].

The role of the welfare state in the social shaping of health has been a widely discussed topic in comparative public health studies in recent decades. However, comparative studies of European welfare regimes at the national level to date do not provide conclusive evidence on the relationship between health inequalities and the implementation of policies oriented towards the universalisation of services, income guarantees and the provision of more generous social transfers—a model characteristic of the Nordic countries [12,13,14]. Given this inconclusive evidence, some researchers suggest that analyses should focus on specific policy areas at different institutional levels rather than state-level normative typologies [15,16,17].

Within the broader perspective of place-based policy and the local production of health inequalities, local governments are receiving increasing attention in analysing health inequalities due to their proximity and capacity to influence people’s daily lives [18,19]. Municipalities also have certain capacities to formulate and implement contextualised and direct interventions on the social determinants of health [20,21,22]. Nevertheless, many research papers focus on welfare states and health inequalities, but few concentrate on the potential influence of local welfare policies on health inequalities. Focusing on the Spanish case, this paper aims to analyse the influence of local policies according to the orientation of municipal spending on the magnitude of health inequalities.

The starting assumption for the present study is that if local governments focus their efforts on redistributive policies (which can affect the social determinants of health), they will affect health inequalities, promoting differences between municipalities and their residents. Thus, local welfare systems could have a moderating effect by acting on health determinants [17]. This approach provides an opportunity to analyse variations in welfare policy efforts and their impacts within the same country. It also opens the way to examining the role of local welfare systems in achieving national welfare system functions. More specifically, our main objective is to analyse the effect of local policy agendas on population health inequalities among Spanish municipalities.

## 2. Local Welfare Policies and Health Inequalities

Analyses of socio-spatial inequalities have shown that inequalities persist, both between and within municipalities, and may be related to the importance of welfare policies in a country [10,23]. Comparative analyses of residential segregation and socio-spatial inequalities in large cities or metropolitan areas show that in addition to the importance of exposure to economic globalisation through the internalisation of local economies, residential segregation levels vary according to welfare regimes and the role of local government in them [11,24,25,26]. The socio-spatial effects of welfare estates illustrate, first, how relevant it is to consider their multi-scale nature; second, the fact that welfare policies can be promoted and implemented by different institutional actors; and third, the role played by local governments [9,27]. These ideas have promoted a research agenda about the role of local governments as welfare provisors and specifically on local welfare systems [4,28].

To a large extent, this line of research began with studies on the northern European universalist welfare regime model. Relevant work has examined municipal governments’ role in adjusting the orientation of the welfare regime toward socio-spatial inequalities promoting differences at this governmental scale [29]. As Sellers and Lindstrom point out, compared with other countries, municipalities in these contexts combine a high level of institutional capacity (competencies, budget, staff, etc.) with a low level of supra-municipal level supervision [30]. This institutional context ensures higher service provision possibilities than other models combining lower levels of local capacity with higher levels of supra-municipal supervision (the corporatist model of continental Europe) or lower levels in both dimensions (the liberal regimes in Anglo-Saxon countries). However, analyses of municipal welfare policies in the universalistic model show that such a combination could result in significant differences in the strength of welfare policies and their impact on inequalities. Therefore, the existence of multiple local welfare systems [31,32,33].

Based on these previous studies, the comparative analysis of local welfare systems has shown the necessity to consider both the model of integration of local governments in each country (or welfare system) and the socio-economic and cultural characteristics of local contexts. This approach is essential to understand how welfare services are provided locally and their effects on inequality processes in municipalities [28]. First, previous research shows local welfare systems adopt different forms according to intergovernmental institutional context, inequalities in municipalities and the policy processes that shape local government decisions concerning the provision of welfare services (the actors who channel demands, local political culture, the ideological orientation of local authorities, …). Second, decisions and strategies adopted by local governments to provide services—given the conditions socio-economic and cultural in each local community—can influence social inequalities within and between municipalities [24,25]. 

As regards the effect of local welfare systems on social inequalities, most studies have focused on policies linked to social integration processes, such as social assistance, income or labour integration programmes [10,34,35,36]; however, less attention has been paid to health. Empirical evidence suggests that individual socio-economic conditions are the root cause of the persistence of large health disparities [37]. The persistent relationship between socio-economic status and health has been widely documented, and a clear social gradient has been identified for multiple health outcomes. We know that poverty and social and economic circumstances affect health throughout the life course, particularly in critical periods linked to different life transitions. People who are lower down on the social ladder are generally at a greater risk of developing severe illnesses and suffering premature death [38]. Far from diminishing or disappearing, health inequities appear to be increasing today in Europe, despite universal access to health services and welfare policies [39,40].

Comparative studies at the national level show no clear relationship between welfare state models and reduced health inequalities; at best, the evidence is contradictory [14,16]. However, as in other inequality processes, the reasons may lie—at least in part—in the effects deriving from the contexts closest to individuals and the actions undertaken by local governments. Previous studies have shown the key role of the local context (the municipality and the neighbourhood) in explaining health inequalities. In addition to individual factors linked to social status or position, the physical characteristics, socio-economic processes or lifestyles that characterise each local community may produce contextual effects on health inequalities [41,42]. This process leads to apparent nationwide differences and health inequalities between socio-spatial contexts [43].

In addition, some studies have demonstrated a relationship between local government spending on health and the improvement of specific health outcomes for the population or reduced inequalities between social groups [44,45]. However, welfare policies, even if not explicitly directed toward reducing such inequalities, can affect the processes and structural causes that condition them [46], as research on area-based initiatives has also shown [47,48]. Studies have also documented that social spending at the local level is associated with a lower risk of mortality in the US [49], especially in the case of social programme spending and potentially preventable mortality [50]. In Europe, most empirical analyses have nevertheless focused on evaluating specific interventions centred on health promotion and education in urban areas [51]. There is still a lack of studies that assess, using a comparative approach, the potential influence of municipal policies on social determinants and health inequalities, depending on the priority given to specific areas of public policy.

## 3. The Institutional ‘Imbrication’ Thesis and the Moderating Role of Local Welfare Policies on Health Inequalities: The Spanish Case

According to the institutional theory proposed by Beckfield et al., welfare policies can influence health inequality determinants through four mechanisms [17]. First, a redistributive mechanism through the transfer of resources between groups (e.g., the redistribution of wealth or income). Second, a regulatory mechanism (compression sets) is based on facilitating access to health services (the direct provision of health services). Third, a mechanism related to a mediation role can be exercised by other determinants or public policies (such as education policies). Fourth, a mechanism relating to the effect of institutional overlap; that is, the fact that the actions of different policies and institutions can overlap and ultimately produce and amplify their impact or moderating effect on inequalities. Institutional effects can certainly overlap, acting and operating in multiple domains. Therefore, at various institutional levels, they can have direct consequences on the population’s health, but they can also exercise an indirect influence through socio-economic and social determinants of health [51]. From this viewpoint, local governments could influence health inequalities via the first three mechanisms, thus producing contextual variations in the relationship between welfare and health policies within the same country.

Nevertheless, given the multi-scale nature of local welfare systems, it can be assumed that institutional overlaps also exist. The combined actions of municipal and national welfare policies could partly explain such contextual variations, which would not depend exclusively on policies and interventions in the field of health, but on the welfare policies that affect their determinants, as well as other explanatory inequalities (employment insertion, education, etc.). The assumption of institutional overlap suggests that within the same country and under the same welfare regime, the magnitude of health inequalities could differ among municipalities depending on the nature of the policies they develop. In other words, depending on the strength of the welfare policies reinforcing the redistributive, regulative or mediating mechanisms that—according to the proposal of Beckfield et al. [17]—would explain the relationship between welfare policies and health inequalities.

The local government system in Spain, similar to others in Southern Europe, is characterised by a limited capacity to provide services, as well as a high level of dependence on supra-municipal authorities. Major welfare policies, such as education, employment or health, are mainly in the hands of regional governments [52,53]. This is a clear example of a limited local welfare regime [54,55]. However, local governments have ‘general competencies’ to autonomously promote policies and provide services in different fields of public policy, including municipal implementation strategies for health prevention or promotion policies, as well as the development of local health schemes [46,47]. Above all, Spanish municipalities play a relevant role as social policy agents for vulnerable groups through social services. All municipalities with over 20,000 inhabitants are responsible for such a policy, as established in the Law Establishing the Local Regime in Spain (Ley 7/1985, de 2 de abril, Reguladora de las Bases de Régiemn Local; Ley 27/2013, de 27 de diciembre, de racionalización y sostenibilidad de la Administración Local) and as implemented in the Concerted Social Services Plan (1988). The management and provision of these services at the municipal level are common in Southern European countries [10,55,56,57,58]. It has been found that welfare spending in Spanish municipalities has some effect on social inequalities [59,60]. Therefore, the Spanish case could be an ‘exemplary case’ to test the institutional overlap thesis regarding municipal welfare policies representing a layer of action taken by other administrations to reduce inequalities between groups.

In line with studies on local welfare systems and the institutional theory on the effects of welfare policies on health distribution, the present work proposes that local welfare policies may have a moderating effect on health inequalities. This effect would allow us to explain some of the municipal variations in health inequalities between different socio-economic groups in Spain. Therefore, our objective is to examine whether a greater orientation of local policies towards certain public policy domains would explain some contextual variations in health inequalities, regardless of municipalities’ socio-economic characteristics. Specifically, we set out to test the following hypotheses:

**H1.**  *A greater orientation of local governments towards redistributive policies is associated with population health improvements in these municipalities*.

**H2.**  *A greater orientation towards redistributive policies moderates the relationship between socio-economic status and health, resulting in lower health inequalities in these municipalities*.

## 4. Methodology

### 4.1. Data

To maximise the municipal sample available, Spain’s 2006–2007 and 2011–2012 National Health Surveys (ENSE) were used as a pooled dataset, with information from both waves. These surveys provide socio-epidemiological information representative of the non-institutionalised adult population in Spain. The respondents were selected using three-stage stratified sampling methods. In the first stage, census strata sections were chosen according to the municipality’s size. Second-stage units were the main family homes. In the third stage, an adult (aged 15 years and over) was selected from each household to complete a questionnaire. The data were collected via face-to-face interviews. The chosen sample corresponded to adults residing in municipalities with populations of over 20,000 inhabitants, in which—as mentioned earlier—municipal governments have to provide social services. Altogether, the sample covered 373 municipalities and 31,378 individuals.

### 4.2. Dependent Variables

Self-perception of general health status, an indicator of healthy practices over the 12 months before the survey and limitations in usual activities due to health problems were used as dependent variables. The first was obtained via the question, “In the last twelve months, would you say that your state of health has been very good, good, not that good, bad or very bad?” The original answers were recoded into two categories: 1 = Good health (very good or good) and 0 = Bad health (not that good, bad or very bad). The second dependent variable was based on multiple questions about specific health-related practices. This indicator was calculated by adding one point for the fulfilment of each of the following practices: intense physical activity two or more times a week, moderate physical activity three or more times a week, dental check-up in the preceding 12 months and healthy diet based on a healthy eating index. In addition, one point was subtracted for each of the following conditions: being overweight or obese, a smoker, consuming sugary soft drinks daily and consuming sweets daily. An indicator with a range of values between −4 and 4 was finally obtained, with the highest values indicating greater adoption of healthier practices. Finally, we use Activity limitations due to health problems as a dependent variable. The question “To what extent have you been limited due to a health problem in performing activities that people usually do (during the last 6 months)?” was recoded into two categories: 0 = No limitations and 1 = Limited or severely limited.

### 4.3. Independent Variables

In order to measure the orientation of local government policies towards different public policy domains, an indicator was developed based on factorial analysis of the expenditure in different policy sectors according to the consolidated budgetary information available from the Ministry of Finance (see supplementary material). This factorial analysis included the percentage of expenditure by six policy areas of the total municipal expenditure for the period 2002–2008, thus avoiding possible spending fluctuations over specific years [60]. It was decided to intentionally select a period for the analysis of expenditure orientation before the health surveys to clarify the temporal sequence between the independent and dependent variable, as well as to consider a “window” of observation in which potential institutional influences on health outcomes were plausible in temporal terms. The six areas considered were: (1) general administration spending; (2) redistributive spending (social protection, social promotion, health, education, other social services, community and housing-urbanism); (3) spending on culture; (4) public spending on citizen security; (5) spending on basic urban services (waste, sewage, cleaning, supply and distribution of water, etc.); and (6) economic development (basic infrastructure, transport, communications, etc., general economic regulation and economic regulation of production sectors). The first factor, explaining approximately 36 per cent of the variance, resembles the classical distinction between development and redistributive policies [61,62]. It identified municipalities with expenditures oriented more towards redistributive policies and less towards economic development, characterised by a low redistributive impact on resident income [59]. Factor scores ranged from −2.13 to 1.01, with higher scores indicating a greater orientation towards redistributive policy areas than towards economic development policies.

The Socio-economic Vulnerability Index (ISVUN-SE), an indicator of residential segregation according to socio-economic status and the Gini coefficient were used as control variables at the municipal level. The ISVUN-SE index was obtained from the Urban Vulnerability Atlas prepared by the Spanish Ministry of Development (2015) based on data from the 2001 Population and Housing Census. This index is based on a multi-criteria national classification of census sections according to specific indicators.

The ISVUN-SE approximates a municipality’s socio-economic vulnerability based on five indicators: the percentage of the unemployed population, the percentage of unemployed youths, the percentage of temporarily employed, the percentage of unskilled employed people and the percentage of the population with no education. Therefore, the indicators measure a set of structural determinants linked to health inequalities that, as mentioned above, usually explain socio-spatial variations. Both synthetic indices vary between 0 (least vulnerable) and 1 (most vulnerable). The residential socio-economic segregation was calculated through the isolation index [63,64], which measures the probability that an individual with low education shares his or her spatial unit of residence (census tract) with an individual of the same group. This indicator was calculated with data from the 2011 Population and Housing Census. Its maximum value would mean that the population with low education is isolated in the units where it resides. Therefore, higher scores indicate a higher level of residential segregation of the low-educated population within the municipality. The Gini index for Spanish municipalities with more than 5000 inhabitants was obtained for 2007 from the database of the Fundación de Estudios de Economía Aplicada (FEDEA), which makes an estimate based on tax data for the municipalities of the common tax system in Spain [65]. Higher values of the Gini index indicate greater income inequality at the municipal level.

At the individual level, in addition to the interviewees’ gender, age, marital status and employment situation, we also considered social class based on the occupation of the reference person in the household. Specifically, social class was operationalised using three groups based on the adaptation of the Goldthorpe scheme proposed by the Spanish Society of Epidemiology [66]: Upper Class (groups I and II), Middle Class (III and IV) and Lower Class (V and VI). Table 1 shows the description of the variables used.

### 4.4. Analysis

To show the contextual effect of the redistributive orientation of local policies on health inequalities, multilevel regression analyses were conducted, in which individuals were grouped in their municipalities of residence (N_municipalities_ = 373). A five-step analysis strategy was followed: in the first step, we estimated an empty model that only included the intersection and random effects at the municipal and individual level (Model 0, shown in the supplementary material); this model allowed us to explore the variance of each dependent variable at the municipal level. In the second step, respondents’ age, gender, marital status, main economic activity, year of survey and social class were added. The objective here was to explore health inequalities according to the individual’s social class. In the third step, contextual variables at the municipal level were included to explore whether they had any direct influence on population health. Fourth, in order to test whether a temporal change in population health could be verified as a function of local policy orientations, an interactive effect was introduced between the municipal expenditure orientation indicator and the survey period (see complete models in the supplementary material). Lastly, in order to test whether local policy orientation could affect the health of various socio-economic groups differently, an interactive effect was introduced between spending orientation and social class. This allowed us to uncover whether the differences between social classes regarding health status, lilmitations and healthy practices diminished when the local spending orientation was more redistributive, regardless of the municipality’s level of vulnerability or other individual variables. The analyses were conducted using the statistical package MLwiN 2.32. Multilevel logistic regression models were applied for the self-perceived good health and activity limitations due to health problems indicators. Linear regression models were used for the healthy practices indicator. We used restricted iterative generalised least squares (RIGLS) and marginal quasi-likelihood (MQL) for the healthy practices indicator and MQL and the penalised quasi-likelihood PQL for the logit models.

## 5. Results

### 5.1. Socio-Economic Gradient in Health

Model 1 (Table 1, Table 2 and Table 3) shows a clear socio-economic gradient for self-perceived good health, healthy practices and activity limitations due to health problems. People in the middle-class category (groups III and IV) were less likely to report being in good health (Logit = −0.452, *p* < 0.001), more likely to report limitations of daily activities (Logit = 0.229, *p* < 0.001) and less likely to adopt healthy practices (β = −0.289, *p* < 0.001) than people in the upper classes (groups I and II). These differences were even greater between the lower classes (groups V and VI) and the upper classes for being in good health (Logit = −0.758, *p* < 0.001), activity limitations (Logit = 0.369, *p* < 0.001) and healthy practices (β = −0.466, *p* < 0.001).

We observed the expected associations based on prior knowledge for the rest of the control variables at the individual level (see complete models in supplementary material). The probability of enjoying good health decreased as age increased (Logit = −0.032, *p* < 0.001), and older people tended to adopt slightly healthier practices (β = 0.005, *p* < 0.001) and suffer more limitations to their daily activities due to health problems (Logit = 0.023, *p* < 0.001). Women reported worse health status (Logit = −0.422, *p* ≤ 0.001) and more activity limitations than men (Logit = 0.374, *p* < 0.001) but adopted healthier practices (β = 0.288, *p* < 0.001). Divorced and separated people presented worse health indicators than married people, as did the unemployed compared with employed people. Regarding the comparison between the two periods studied, the indicators seem to present a positive evolution: people interviewed in 2011–2012 report more probability of having good health (Logit = 0.394, *p* < 0.001), fewer limitations of daily activity (Logit = −0.290, *p* < 0.001) and higher score in the healthy practices index (β = 0.466, *p* < 0.001) than people interviewed in 2006–2007.

### 5.2. Local Contextual Effects

Model 2 includes contextual indicators at the municipal level. The results show a negative association between the socio-economic vulnerability of the municipality (ISVUN-SE) and self-perceived health. This association suggests that people residing in more vulnerable municipalities are less likely to report good health regardless of their individual characteristics (Logit = −0.789; *p* < 0.05). However, no significant association was observed between the socio-economic vulnerability index and the probability of suffering limitations of daily activity due to health problems, as well as in the case of the adoption of healthy practices. Moreover, no significant association was observed between indicators of residential segregation and income inequality at the municipal level and the health outcomes analysed.

The results show no significant association between the orientation of redistributive spending at the municipal level and the health outcomes analysed for the population as a whole. However, the results suggest that there is a trend of improvement in the proportion of people reporting good health in those municipalities where there has been higher redistributive spending; the effect is observable due to the inclusion of the interaction between these two variables in model 2a of the supplementary material. This result suggests that residents in municipalities where spending was more oriented towards redistributive policies seemed to improve their health more during 2011–2012 compared to 2006–2007 (Logit = 0.183; *p* < 0.1). However, this effect was not visible in the case of activity limitations and healthy practices. Therefore, confirming this period effect would require further extension of the analyses with the inclusion of new waves of the surveys used to lengthen the analysis periods.

### 5.3. Moderating Effects of Local Policies

**Figure 1:**
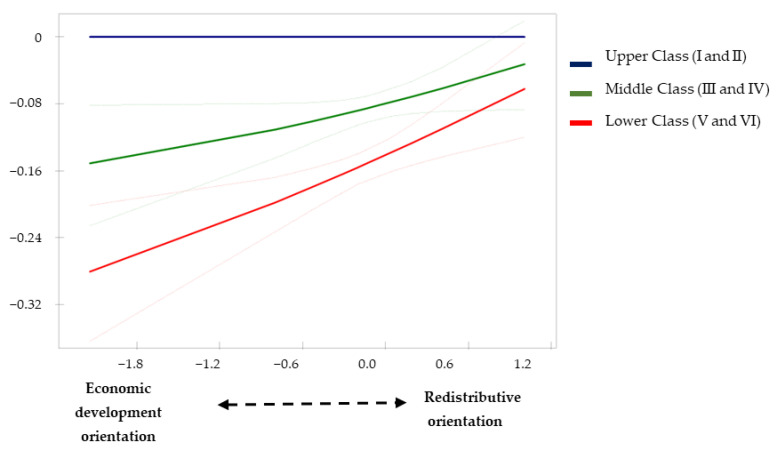
Differences in health status compared with Upper Class (I and II) according to local spending orientation (Model 3, Table 2).

**Figure 2:**
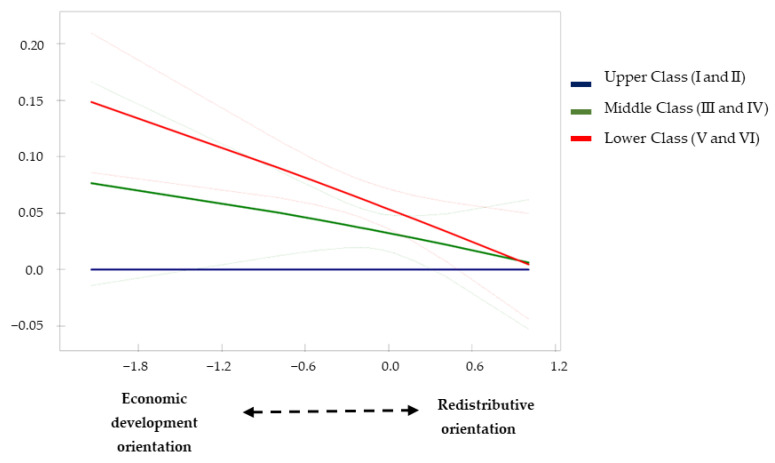
Differences in the activity limitations due to health problems compared with Upper Class (I and II) according to the redistributive orientation of local spending (Model 3; Table 3).

**Figure 3:**
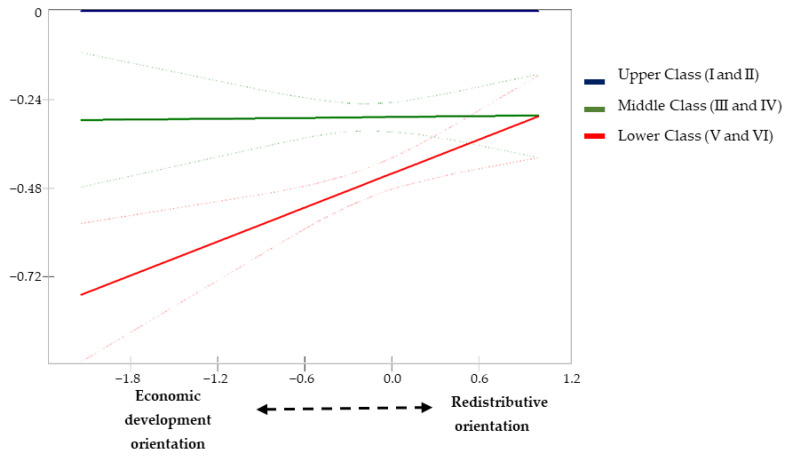
Differences in the adoption of healthy practices compared with Upper Class (I and II) according to the redistributive orientation of local spending (Model 3; Table 4).

In Model 3, an interactive effect between social class and our expenditure orientation indicator was introduced in order to explore possible moderating effects on health inequalities (Table 2, Table 3 and Table 4). The results suggest that middle- and lower-class people are more likely to report good health when their municipalities of residence focus their spending efforts on redistributive policy areas rather than economic development policies (Table 2, Model 3: Redistributive Orientation*Middle Class: Logit = 0.250, *p* < 0.05, and Redistributive Orientation*Lower Class: Logit = 0.393, *p* < 0.001). The predicted probabilities from this model suggest that the redistributive orientation of local policies contributes to reducing social class differences in good health (Figure 1). The results also show that people of lower social class reduce their probability of suffering limitations in daily activity when they reside in municipalities whose spending effort is oriented towards redistributive policies (Table 3, Model 3: Redistributive Orientation*Lower Class: Logit = −0.284, *p* < 0.05). As illustrated in Figure 2, the predicted probabilities also indicate a reduction in class inequalities in activity limitations due to health problems as the orientation of redistributive spending at the local level increases. Finally, the results of model 3 in Table 4 suggest the same pattern for the adoption of healthy practices by people from lower social classes (Redistributive Orientation*Lower Class: β = 0.157, *p* < 0.001). Figure 3 illustrates how differences in the likelihood of adopting healthy practices are significantly reduced between the lower and upper classes when the orientation of local spending is towards redistributive areas rather than economic and development policy areas.

## 6. Discussion

Health inequalities persist despite major changes in the prevalence and incidence of diseases and their associated risk factors [37,67,68]. Welfare policies have been conceptualised by social epidemiology as important determinants of health [16]. Previous studies support the idea that welfare state generosity can have a positive impact on the health of the population as a whole [69]. Scandinavian and Anglo-Saxon welfare regimes have been documented to have better overall self-perceived health than Bismarckian, Southern and Eastern European welfare regimes [70]. However, comparative studies in Europe provide inconclusive and mixed evidence on the ability of welfare states characterised by policies oriented towards universalisation of services, income guarantees and more generous provision of social transfers to reduce health inequalities [14,40,71]. This mixed evidence raises the question of whether state policies aimed at improving health and living conditions are sufficient to reduce health inequality and limit its reproduction over time. This paper supports the idea that a multi-scalar approach to welfare policy analysis is needed to clarify whether or not welfare provisions have an impact on health inequalities.

In relation to our first hypothesis, our results do not support that a greater orientation of local policy towards redistributive policies achieves overall improvements in the health of the municipality’s residents as a whole. However, our findings do support the idea that such an orientation towards redistributive areas of local policy does reduce health inequalities between those with lower and higher socio-economic status. Our findings suggest that local government policies can explain contextual variations in the determinants of health inequalities within the same welfare model or regime regardless of the socio-economic characteristics of the municipalities (measured here in terms of socio-economic vulnerability, socio-economic residential segregation and income inequality). In other words, the action of local governments on the determinants of health inequalities has a moderating effect on them; specifically, this local action ‘moderates’ the effect of social class on health inequalities.

As mentioned, previous studies have shown the importance of considering the sub-national level of government to understand the effects of welfare policies. Our results confirm this, thereby making an interesting contribution to the role of local welfare systems in improving citizens’ quality of life, analysed here as health conditions—specifically, its impacts on health inequalities. As indicated above, the case of Spain is a typical example of a welfare system in which municipalities have limited capacity to provide welfare services. Accordingly, the effect of the institutional overlapping mechanism may be more limited than in welfare regimes in which the municipalities have more capabilities to provide welfare services, such as in northern Europe. Even so, the results show that municipal action can moderate the effect of social class on health. In this way, in line with the ‘institutional imbrication’ approach of Bekfield et al. [17], our results allow us to infer that different policies and different institutional settings produce a joint action around the provision of welfare services, contributing to the reducing inequalities and improving citizens’ quality of life.

This represents a relevant contribution to the study of local welfare systems, confirming their key role in social inequalities and emphasising the need to analyse existing variations within the same model or welfare regime. Thus, the present study opens up an interesting line of research: the analysis of variations in different areas of inequality within the same country or welfare model according to differences in local governments’ actions. Here, the effect of local welfare systems, as spending orientation, on health inequalities has been studied.

The approach adopted in the present work allowed us to address the analysis of health inequalities in a novel way by considering the contextual variations resulting from the orientation of municipal agendas towards redistributive policies. On the one hand, we found that greater redistributive spending was associated with health status improvements for residents. This made it possible to infer that, as was advanced in the hypotheses, a moderating effect of local welfare systems existed on health due to the impact of these policies on the social determinants of health. However, this result also contributes to studies of the effects of local welfare systems on health. These studies focus mainly on national welfare system characteristics that do not permit the estimation of potential variations within the same model [14,15]. Our approach could be reproduced in international comparative studies, acknowledging the role of local welfare systems on health.

Our analysis studies the action of local governments based on their spending orientation. We did not consider other aspects of the action taken by local governments or other actors as welfare providers at the local level that have been highlighted in prior literature about local welfare systems. These aspects could also shed light on the moderating effects presented here, in line with the comparative studies in different European cities mentioned above, where other policy sectors are examined in greater depth [10,36]. Nevertheless, our work offers additional evidence of the impact of local welfare systems on inequalities in an under-explored field such as health.

## 7. Conclusions

The results of this study suggest that the relationship between welfare policies and health inequalities at the national level can vary depending on the contextual effects deriving from actions by local or sub-national governments. As might be expected, we have observed that the magnitude of such effects is less than that derived from an individual’s social position. They point, however, to the opportunity of exploring the institutional overlap thesis to improve our understanding of this relationship within the growing research agenda on the role of sub-national governments in welfare systems based on comparative studies. It would therefore be relevant to advance possible lines of work and methodologies to analyse the welfare state’s multi-scalar nature and the form it takes in each country to analyse local variations, as well as the importance of these variations to compare the effects of different welfare regimes on inequalities reduction.

## Figures and Tables

**Table 1:** Descriptors of the variables included in the analyses.

Dependent Variables					
		Yes		No	
Good health (%)		70		30	
		Mean	SD	Min.	Max.
Healthy Practices Indicator		−0.57	1.28	−4.00	4.00
Activity limitations due to health problems (%)		21.2		78.8	
Independent Variables					
Municipalities		Mean	SD	Min.	Max.
Redistributive orientation		0.17	0.43	−2.13	1.01
Socio-economic Vulnerability Index (ISVUN-SE)		0.57	0.10	0.27	1.00
Residential isolation index		0.19	0.07	0.06	0.44
Gini index at the municipal level		0.45	0.04	0.33	0.64
Individuals		Mean	SD	Min.	Max.
Age (mean, SD)		46	18.43	15.00	104.00
Period (%)	2006–2007	49.00			
2006–2007	2011–2012	51.00			
Gender (%)	Man	49.00			
	Woman	51.00			
Marital status (%)	Married	55.07			
	Single	33.19			
	Widow/widower	6.96			
	Separated	2.16			
	Divorced	2.61			
Economic Activity (%)	Working	48.25			
	Unemployed	11.13			
	Retired	19.61			
	Studying	8.26			
	Homemaker	12.03			
	Other	0.71			
Social Class (%)	Upper Class: I and II	21.20			
	Middle Class (III and IV)	42.60			
	Lower Class (V and VI)	33.50			

**Table 2:** Multilevel models for self-perceived good health.

	Model 0			Model 1			Model 2			Model 3		
	Logit	SE		Logit	SE		Logit	SE		Logit	SE	
Fixed Part												
Cons	0.814	(0.022)	***	1.536	(0.047)	***	1.553	(0.046)	***	1.555	(0.051)	***
Social Class (Ref. Upper Class: I and II)												
Middle Class (III and IV)				−0.452	(0.037)	***	−0.444	(0.038)	***	−0.441	(0.041)	***
Lower Class (V and VI)				−0.758	(0.046)	***	−0.738	(0.048)	***	−0.738	(0.045)	***
Period 2011–2012 (ref. Period 2006–2007)				0.394	(0.043)	***	0.394	(0.044)	***	0.394	(0.044)	***
Socio-economic Vulnerability Index (ISVUN-SE)							−0.789	(0.352)	*	−0.776	(0.356)	*
Residential isolation index							−0.217	(0.775)		−0.289	(0.784)	
Gini index at the municipal level							0.943	(0.658)		0.994	(0.657)	
Redistributive orientation							−0.022	(0.057)		−0.375	(0.117)	**
Redistributive orientation*Period 2011–2012										0.151	(0.100)	
Redistributive orientation*Middle Class (III and IV)										0.250	(0.110)	*
Redistributive orientation*Lower Class (V and VI)										0.393	(0.111)	***
Random Part												
Municipal level	0.077	(0.010)		0.088	(0.013)		0.073	(0.011)		0.073	(0.011)	
Individual level	3.290			3.290			3.290			3.290		
VPC												
Municipal level	2.3%			2.6%			2.2%			2.2%		
Individual level	97.7%			97.4%			97.8%			97.8%		
N municipalities	373			373			373			373		
N individuals	30,279			30,279			30,279			30,279		

*** *p* < 0.001; ** *p* < 0.01; * *p* < 0.05 VPC = Variance partition coefficient. SE = Standard Error. All analyses were controlled for age, gender, marital status and the relationship of the individual to economic activity (see full models in Supplementary Materials).

**Table 3:** Multilevel models for activity limitations due to health problems.

	Model 0			Model 1			Model 2			Model 3		
	Logit	SE		Logit	SE		Logit	SE		Logit	SE	
Fixed Part												
Cons	−1.322	(0.026)	***	−1.865	(0.058)	***	−1.877	(0.057)	***	−1.879	(0.059)	***
Social Class (Ref. Upper Class: I and II)												
Middle Class (III and IV)				0.229	(0.051)	***	0.224	(0.051)	***	0.224	(0.055)	***
Lower Class (V and VI)				0.369	(0.049)	***	0.363	(0.050)	***	0.365	(0.051)	***
Period 2011–2012 (ref. Period 2006–2007)				−0.290	(0.051)	***	−0.290	(0.051)	***	−0.291	(0.051)	***
Socio-economic Vulnerability Index (ISVUN-SE)							0.007	(0.390)		0.004	(0.390)	
Residential isolation index							0.577	(0.921)		0.634	(0.917)	
Gini index at the municipal level							−1.260	(0.711)		−1.269	(0.709)	
Redistributive orientation							0.005	(0.060)		0.146	(0.114)	
Redistributive orientation*Period 2011–2012										0.066	(0.104)	
Redistributive orientation*Middle Class (III and IV)										−0.153	(0.156)	
Redistributive orientation*Lower Class (V and VI)										−0.284	(0.113)	*
Random Part												
Municipal level	0.106	(0.014)		0.113	(0.016)		0.111	(0.015)		0.11	(0.015)	
Individual level	3.290			3.290			3.290			3.290		
VPC												
Municipal level	3.1%			3.3%			3.3%			3.2%		
Individual level	96.9%			96.7%			96.7%			96.8%		
N municipalities	373			373			373			373		
N individuals	30,276			30,276			30,276			30,276		

*** *p* < 0.001; * *p* < 0.05. VPC = Variance partition coefficient. SE = Standard Error. All analyses were controlled for age, gender, marital status and the relationship of the individual to economic activity (see full models in supplementary material).

**Table 4:** Multilevel models for healthy practices.

	Model 0			Model 1			Model 2			Model 3		
	β	SE		β	SE		β	SE		β	SE	
Fixed Part												
Cons	−0.571	(0.016)	***	−0.670	(0,029)	***	−0.653	(0.028)	***	−0.653	(0.028)	***
Social Class (Ref. Upper Class: I and II)												
Middle Class (III and IV)				−0.289	(0,018)	***	−0.290	(0.018)	***	−0.288	(0.019)	***
Lower Class (V and VI)				−0.466	(0,023)	***	−0.466	(0.023)	***	−0.465	(0.022)	***
Period 2011–2012 (ref. Period 2006–2007)				0.466	(0,030)	***	0.452	(0.029)	***	0.452	(0.029)	***
Socio-economic Vulnerability Index (ISVUN-SE)							−0.226	(0.189)		−0.218	(0.188)	
Residential isolation index							−0.198	(0.461)		−0.241	(0.460)	
Gini index at the municipal level							0.453	(0.423)		0.454	(0.423)	
Redistributive orientation							0.022	(0.035)		−0.023	(0.053)	
Redistributive orientation*Period 2011–2012										−0.047	(0.069)	
Redistributive orientation*Middle Class (III and IV)										0.006	(0.041)	
Redistributive orientation*Lower Class (V and VI)										0.157	(0.045)	***
Random Part												
Municipal level	0.058	(0.007)		0.046	(0.006)		0.046	(0.006)		0.046	(0.006)	
Individual level	1.599	(0.024)		1.487	(0.024)		1.491	(0.025)		1.490	(0.025)	
VPC												
Municipal level	3.5%			3.0%			3.0%			3.0%		
Individual level	96.5%			97.0%			97.0%			97.0%		
N municipalities	373			373			373			373		
N individuals	30,263			30,263			30,263			30,263		
−2*loglikelihood:	109,577.845			106,874.17			103,556.963			103,538.195		

*** *p* < 0.001;. VPC = Variance partition coefficient. SE = Standard Error. All analyses were controlled for age, gender, marital status and the relationship of the individual to economic activity (see full models in Supplementary Materials).

## Data Availability

Restrictions apply to the availability of these data. Data were obtained from the Instituto Nacional de Estadística through a specific agreement. This agreement limit data sharing.

## Supplementary Materials

The following supporting information can be downloaded at: https://www.mdpi.com/article/10.3390/ijerph192315447/s1, Table S1: Factorial anal Factorial analysis of the orientation of municipal government expenditure in Spain (2002–2008); Table S2: Complete multilevel logistic regression models for self-perceived good health; Table S3: Complete multilevel logistic regression models for Activity limitations due to health problems; Table S4: Complete multilevel linear regression models for healthy practices.
