# Supplementary material for: Local Welfare Systems and Health Inequalities: The Effects of Institutional Overlapping and Local Variations
Source: Int J Environ Res Public Health. 2022 Nov 22;19(23):15447. doi: 10.3390/ijerph192315447 (PMC9739049; doi:10.3390/ijerph192315447)
Supplement: Supplementary file 1 [file ijerph-19-15447-s001.zip › ijerph-1956454-supplementary.pdf]

Local welfare policies and health inequalities. The effects of institutional overlapping and local variations

María Jesús Rodríguez-García, Clemente J. Navarro-Yáñez and Ángel Ramón Zapata-Moya

Supplementary materials

Table S1. Factorial analysis of the orientation of municipal government expenditure in Spain (2002–2008)

**Percentage of local spending on different policy areas**	**Descriptives**		**Factor analysis (1st component)**	**Factor analysis (1st component)**
**Average % 2002-2008 period**	Mean	Std. Dev.	1	2
General administration	0.25	0.15	-0.20	-0,89
Redistributive	0.32	0.13	**0.70**	0,33
Culture	0.11	0.07	0.48	0,49
Public safety	0.02	0.03	-0.07	0,64
Basic urban	0.11	0.08	0.49	0,08
Developmental expenditures	0.15	0.14	**-0.90**	0,23
Explained variance (%)			36.08	20,97
Barlett sphericity			Chi2-20206.4189964093<br>gl=15; Sig.=0.000	

N=7678 Spanish municipalities. Extraction method: Principal Component Analysis. Rotation method: Varimax with Kaiser Normalization.

Table S2. Complete multilevel logistic regression models for self-perceived good health

	Model 0				Model 1				Model 2				Model 2a				Model 3			
	Logit	SE	OR		Logit	SE	OR		Logit	SE	OR		Logit	SE	OR		Logit	SE	OR	
**Fixed Part**																				
Cons	0.814	(0.022)	2.26	***	1.536	(0.047)	4.65	***	1.553	(0.046)	4.73	***	1.553	(0.047)	4.73	***	1.555	(0.051)	4.74	***
Age					-0.032	(0.002)	0.97	***	-0.032	(0.002)	0.97	***	-0.032	(0.002)	0.97	***	-0.032	(0.002)	0.97	***
Women (ref. Men)					-0.422	(0.037)	0.66	***	-0.426	(0.038)	0.65	***	-0.425	(0.038)	0.65	***	-0.423	(0.038)	0.66	***
Marital status (ref. Married)																				
Single					-0.043	(0.046)	0.96		-0.051	(0.047)	0.95		-0.052	(0.047)	0.95		-0.054	(0.047)	0.95	
Widowed					0.019	(0.050)	1.02		0.022	(0.052)	1.02		0.019	(0.052)	1.02		0.020	(0.052)	1.02	
Separated					-0.193	(0.091)	0.82	*	-0.168	(0.093)	0.85	+	-0.170	(0.093)	0.84	+	-0.172	(0.093)	0.84	+
Divorced					-0.421	(0.087)	0.66	***	-0.408	(0.090)	0.66	***	-0.408	(0.090)	0.66	***	-0.407	(0.090)	0.67	***
Economic Activity (ref. Working)																				
Unemployed					-0.404	(0.064)	0.67	***	-0.404	(0.064)	0.67	***	-0.405	(0.064)	0.67	***	-0.405	(0.064)	0.67	***
Retired					-0.691	(0.060)	0.50	***	-0.682	(0.062)	0.51	***	-0.682	(0.062)	0.51	***	-0.683	(0.062)	0.51	***
Student					0.403	(0.111)	1.50	***	0.404	(0.113)	1.50	***	0.404	(0.113)	1.50	***	0.406	(0.113)	1.50	***
Housework					-0.433	(0.052)	0.65	***	-0.417	(0.054)	0.66	***	-0.420	(0.054)	0.66	***	-0.422	(0.054)	0.66	***
Others					-0.879	(0.171)	0.42	***	-0.845	(0.176)	0.43	***	-0.852	(0.177)	0.43	***	-0.858	(0.177)	0.42	***
Social Class (Ref. Upper Class: I and II)																				
Middle Class (III and IV)					-0.452	(0.037)	0.64	***	-0.444	(0.038)	0.64	***	-0.442	(0.038)	0.64	***	-0.441	(0.041)	0.64	***
Lower Class (V and VI)					-0.758	(0.046)	0.47	***	-0.738	(0.048)	0.48	***	-0.737	(0.048)	0.48	***	-0.738	(0.045)	0.48	***
Period 2011–2012 (ref. Period 2006–2007)					0.394	(0.043)	1.48	***	0.394	(0.044)	1.48	***	0.393	(0.045)	1.48	***	0.394	(0.044)	1.48	***
Socio-economic Vulnerability Index (ISVUN-SE)									-0.789	(0.352)	0.45	*	-0.782	(0.354)	0.46	*	-0.776	(0.356)	0.46	*
Residential isolation index (people with low education)									-0.217	(0.775)	0.80		-0.222	(0.780)	0.80		-0.289	(0.784)		
Gini index at the municipal level									0.943	(0.658)	2.57		0.960	(0.659)	2.61		0.994	(0.657)		
Redistributive orientation									-0.022	(0.057)	0.98		-0.124	(0.069)	0.88	+	-0.375	(0.117)	0.69	**
Redistributive orientation*Period 2011–2012													0.185	(0.098)	1.20	+	0.151	(0.100)	1.16	
Redistributive orientation*Middle Class (III and IV)																	0.250	(0.110)	1.28	*
Redistributive orientation*Lower Class (V and VI)																	0.393	(0.111)	1.48	***
**Random Part**																				
Municipal level	0.077	(0.010)			0.088	(0.013)			0.073	(0.011)			0.073	(0.011)			0.073	(0.011)		
Individual level	3.290				3.290				3.290				3.290				3.290			
**VPC**																				
Municipal level		2.3%				2.6%				2.2%				2.2%				2.2%		
Individual level		97.7%				97.4%				97.8%				97.8%				97.8%		
N municipalities		373				373				373				373				373		
N individuals		30279				30279				30279				30279				30279		

*** $p < 0.001$; ** $p < 0.01$; * $p < 0.05$; + $p < 0.1$.

Table S3. Complete multilevel logistic regression models for Activity limitations due to health problems.

	Model 0				Model 1				Model 2				Model 2a				Model 3			
	Logit	SE	OR		Logit	SE	OR		Logit	SE	OR		Logit	SE	OR		Logit	SE	OR	
**Fixed Part**																				
Cons	-1.322	(0.026)	0.27	***	-1.865	(0.058)	0.15	***	-1.877	(0.057)	0.15	***	-1.877	(0.057)	0.15	***	-1.879	(0.059)	0.15	***
Age					0.023	(0.002)	1.02	***	0.023	(0.002)	1.02	***	0.023	(0.002)	1.02	***	0.023	(0.002)	1.02	***
Women (ref. Men)					0.374	(0.043)	1.45	***	0.375	(0.043)	1.45	***	0.375	(0.043)	1.45	***	0.375	(0.043)	1.45	***
Marital status (ref. Married)																				
Single					0.104	(0.057)	1.11		0.105	(0.057)	1.11		0.105	(0.057)	1.11		0.106	(0.058)	1.11	+
Widowed					0.077	(0.058)	1.08		0.076	(0.058)	1.08		0.075	(0.058)	1.08		0.074	(0.058)	1.08	
Separated					0.130	(0.107)	1.14		0.130	(0.107)	1.14		0.130	(0.107)	1.14		0.130	(0.107)	1.14	
Divorced					0.406	(0.098)	1.50	***	0.406	(0.098)	1.50	***	0.406	(0.098)	1.50	***	0.405	(0.099)	1.50	***
Economic Activity (ref. Working)																				
Unemployed					0.201	(0.061)	1.22	***	0.200	(0.061)	1.22	**	0.200	(0.061)	1.22	**	0.200	(0.061)	1.22	**
Retired					0.719	(0.068)	2.05	***	0.717	(0.068)	2.05	***	0.717	(0.068)	2.05	***	0.718	(0.068)	2.05	***
Student					-0.270	(0.109)	0.76	*	-0.268	(0.109)	0.76	*	-0.268	(0.109)	0.76	*	-0.268	(0.109)	0.76	*
Housework					0.230	(0.057)	1.26	***	0.227	(0.057)	1.25	***	0.226	(0.057)	1.25	***	0.228	(0.057)	1.26	***
Others					1.323	(0.162)	3.75	***	1.322	(0.162)	3.75	***	1.320	(0.161)	3.74	***	1.325	(0.161)	3.76	***
Social Class (Ref. Upper Class: I and II)																				
Middle Class (III and IV)					0.229	(0.051)	1.26	***	0.224	(0.051)	1.25	***	0.224	(0.051)	1.25	***	0.224	(0.055)	1.25	***
Lower Class (V and VI)					0.369	(0.049)	1.45	***	0.363	(0.050)	1.44	***	0.364	(0.050)	1.44	***	0.365	(0.051)	1.44	***
Period 2011–2012 (ref. Period 2006–2007)					-0.290	(0.051)	0.75	***	-0.290	(0.051)	0.75	***	-0.291	(0.051)	0.75	***	-0.291	(0.051)	0.75	***
Socio-economic Vulnerability Index (ISVUN-SE)									0.007	(0.390)	1.01		0.009	(0.390)	1.01		0.004	(0.390)	1.00	
Residential isolation index (people with low education)									0.577	(0.921)	1.78		0.576	(0.921)	1.78		0.634	(0.917)		
Gini index at the municipal level									-1.260	(0.711)	0.28		-1.257	(0.711)	0.28		-1.269	(0.709)		
Redistributive orientation									0.005	(0.060)	1.01		-0.017	(0.085)	0.98		0.146	(0.114)	1.16	
Redistributive orientation*Period 2011–2012													0.040	(0.107)	1.04		0.066	(0.104)	1.07	
Redistributive orientation*Middle Class (III and IV)																	-0.153	(0.156)	0.86	
Redistributive orientation*Lower Class (V and VI)																	-0.284	(0.113)	0.75	*
**Random Part**																				
Municipal level	0.106	(0.014)			0.113	(0.016)			0.111	(0.015)			0.11	(0.020)			0.11	(0.015)		
Individual level	3.290				3.290				3.290				3.290				3.290			
**VPC**																				
Municipal level		3.1%				3.3%				3.3%				3.2%				3.2%		
Individual level		96.9%				96.7%				96.7%				96.8%				96.8%		
N municipalities		373				373				373				373				373		
N individuals		30276				30276				30276				30276				30276		

*** $p < 0.001$; ** $p < 0.01$; * $p < 0.05$; + $p < 0.1$.

Table S4. Complete multilevel linear regression models for healthy practices

	Model 0			Model 1			Model 2			Model 2a			Model 3		
	β	SE		β	SE		β	SE		β	SE		β	SE	
**Fixed Part**															
Cons	-0.571	(0.016)	***	-0.670	(0.029)	***	-0.653	(0.028)	***	-0.653	(0.028)	***	-0.653	(0.028)	***
Age				0.005	(0.001)	***	0.005	(0.001)	***	0.005	(0.001)	***	0.005	(0.001)	***
Women (ref. Men)				0.288	(0.020)	***	0.290	(0.020)	***	0.290	(0.020)	***	0.290	(0.020)	***
Marital status (ref. Married)															
Single				0.062	(0.023)	**	0.063	(0.023)	**	0.063	(0.023)	**	0.062	(0.023)	**
Widowed				-0.031	(0.028)		-0.033	(0.028)		-0.033	(0.028)		-0.032	(0.029)	
Separated				-0.174	(0.053)	**	-0.166	(0.054)	**	-0.166	(0.054)	**	-0.168	(0.054)	**
Divorced				-0.171	(0.042)	***	-0.167	(0.044)	***	-0.167	(0.044)	***	-0.167	(0.044)	***
Economic Activity (ref. Working)															
Unemployed				-0.130	(0.034)	***	-0.140	(0.034)	***	-0.140	(0.034)	***	-0.139	(0.034)	***
Retired				-0.013	(0.028)		-0.015	(0.029)		-0.015	(0.029)		-0.014	(0.029)	
Student				0.487	(0.046)	***	0.484	(0.048)	***	0.483	(0.048)	***	0.485	(0.048)	***
Housework				-0.043	(0.034)		-0.038	(0.034)		-0.038	(0.034)		-0.037	(0.034)	
Others				0.017	(0.074)		0.031	(0.073)		0.032	(0.073)		0.034	(0.074)	
Social Class (Ref. Upper Class: I and II)															
Middle Class (III and IV)				-0.289	(0.018)	***	-0.290	(0.018)	***	-0.290	(0.018)	***	-0.288	(0.019)	***
Lower Class (V and VI)				-0.466	(0.023)	***	-0.466	(0.023)	***	-0.466	(0.023)	***	-0.465	(0.022)	***
Period 2011–2012 (ref. Period 2006–2007)				0.466	(0.030)	***	0.452	(0.029)	***	0.452	(0.029)	***	0.452	(0.029)	***
Socio-economic Vulnerability Index (ISVUN-SE)							-0.226	(0.189)		-0.228	(0.189)		-0.218	(0.188)	
Residential isolation index (people with low education)							-0.198	(0.461)		-0.196	(0.461)		-0.241	(0.460)	
Gini index at the municipal level							0.453	(0.423)		0.452	(0.424)		0.454	(0.423)	
Redistributive orientation							0.022	(0.035)		0.033	(0.042)		-0.023	(0.053)	
Redistributive orientation*Period 2011–2012										-0.018	(0.067)		-0.047	(0.069)	
Redistributive orientation*Middle Class (III and IV)													0.006	(0.041)	
Redistributive orientation*Lower Class (V and VI)													0.157	(0.045)	***
**Random Part**															
Municipal level	0.058	(0.007)		0.046	(0.006)		0.046	(0.006)		0.046	(0.006)		0.046	(0.006)	
Individual level	1.599	(0.024)		1.487	(0.024)		1.491	(0.025)		1.491	(0.025)		1.490	(0.025)	
**VPC**															
Municipal level	3.5%			3.0%			3.0%			3.0%			3.0%		
Individual level	96.5%			97.0%			97.0%			97.0%			97.0%		
N municipalities	373			373			373			373			373		
N individuals	30263			30263			30263			30263			30263		
-2*loglikelihood:	109577.845			106874.17			103556.963			103556.697			103538.195		

*** p < 0.001; ** p < 0.01; * p < 0.05; + p < 0.1.
